# Movement Retraining and Peak Landing Force, a Modifiable Anterior Cruciate Ligament Injury Risk Marker, in Athletes: A Systematic Review and Meta-Analysis for Primary Prevention

**DOI:** 10.3390/jfmk11030259

**Published:** 2026-06-29

**Authors:** Taeseok Choi, Hanshin Jeong, Yohan Uhm, Yoonhwan Kim

**Affiliations:** Department of Physical Therapy, Kunjang University, Gunsan 54045, Republic of Korea; hsjung@kunjang.ac.kr (H.J.); yheom@kunjang.ac.kr (Y.U.); yhkim1@kunjang.ac.kr (Y.K.)

**Keywords:** plyometric exercise, neuromuscular training, ground reaction force, landing biomechanics, movement retraining, anterior cruciate ligament, injury prevention, athletes

## Abstract

**Background**: Non-contact anterior cruciate ligament (ACL) injury is common and disabling, often requiring reconstruction and predisposing individuals to early post-traumatic osteoarthritis, making scalable, exercise-based prevention a clinical and public health priority. Excessive peak vertical ground reaction force (vGRF) during landing is a modifiable biomechanical risk marker for ACL injury, although whether reducing it lowers injury incidence is unproven. We evaluated the effect of movement retraining on peak vGRF during landing in pivot-sport athletes and general athletic populations. **Methods**: MEDLINE (PubMed), Embase, and the Cochrane Central Register of Controlled Trials were searched from inception through to 25 May 2026. Two reviewers independently screened records and extracted data. Random-effects meta-analyses (DerSimonian–Laird) used Hedges’ g; risk of bias was assessed with RoB 2 and certainty with GRADE. The protocol was registered in PROSPERO (CRD42025116119). **Results**: Nine comparisons from eight randomised controlled trials (292 participants) were included. Movement retraining significantly reduced peak vGRF (Hedges’ g = −0.94, 95% CI −1.34 to −0.54; I^2^ = 63%), with larger effects in general athletic populations (g = −1.50) than in pivot-sport athletes (g = −0.66; subgroup difference *p* = 0.005). Knee flexion angle at initial contact showed a non-significant increasing trend (g = 0.48; *p* = 0.18). Certainty of evidence (GRADE) was low. **Conclusions**: Movement retraining was associated with a reduction in peak vGRF during landing, a surrogate biomechanical marker for ACL injury, on the basis of low-certainty evidence with substantial heterogeneity (I^2^ = 63%). A subgroup difference favouring general over pivot-sport athletes was observed but is exploratory, resting on only three general-athletic comparisons. Because no included trial measured injury incidence, whether these biomechanical changes reduce ACL injury is unknown, and the findings should be regarded as hypothesis-generating.

## 1. Introduction

Anterior cruciate ligament (ACL) injury is a costly and disabling musculoskeletal condition that imposes a substantial clinical and public health burden extending well beyond competitive sport. In the United States alone, over 250,000 ACL reconstructions are performed annually [1], representing a considerable volume of surgical care, prolonged rehabilitation, and lost participation in work, study, and physical activity that recurs as a cost to health systems. Critically, the burden does not end with surgical repair: a high proportion of patients go on to develop post-traumatic knee osteoarthritis within one to two decades of injury, with consequent pain, functional limitation, and reduced health-related quality of life across their lifespan, often in individuals injured in adolescence or early adulthood [2]. The majority of these injuries (approximately 70%) occur through non-contact mechanisms during landing, cutting, and pivoting movements, particularly in sports requiring rapid directional changes such as basketball, soccer, and volleyball [3]; because such non-contact mechanisms are, in principle, modifiable, they represent a tractable target for primary prevention. Female athletes demonstrate substantially higher ACL injury rates—reported as several-fold greater—than male athletes participating in the same sports [4], identifying a clearly defined high-risk population for whom targeted preventive strategies are warranted. These features establish the primary prevention of ACL injury—through scalable, exercise-based interventions deliverable at the population level—as a legitimate preventive medicine and health service priority, with athletes representing an accessible entry point to a condition whose clinical burden extends across the lifespan, while acknowledging that whether modifying landing mechanics ultimately reduces injury or its long-term sequelae remains to be demonstrated.

Vertical ground reaction force (vGRF) during landing has been identified as a critical modifiable biomechanical risk factor for ACL injury [5]. Athletes who subsequently sustain ACL injuries demonstrate an approximately 20% higher peak vGRF during pre-injury screening compared to uninjured controls [6]. Excessive vGRF is directly associated with increased anterior tibial shear forces and dynamic knee valgus moments, both recognised as primary mechanisms underlying non-contact ACL injury [7]. These associations are prospective and biomechanically plausible but do not establish that peak vGRF independently predicts ACL injury or that reducing it lowers injury incidence; vGRF is therefore treated here as a modifiable surrogate marker rather than a validated predictor of clinical injury.

Movement retraining interventions, encompassing plyometric training, neuromuscular training programmes, and landing technique feedback, have emerged as promising strategies to modify high-risk landing biomechanics [8]. These interventions typically focus on enhancing neuromuscular control, optimising movement patterns, and developing proper landing techniques through structured progressive training protocols [9]. Plyometric training specifically targets the stretch-shortening cycle, potentially improving an athlete’s capacity to attenuate vGRF through enhanced eccentric muscle control and improved motor programming [10].

The effectiveness of these interventions may vary across different athletic populations. Pivot-sport athletes, who regularly perform sport-specific landing tasks, may respond differently to movement retraining compared to general athletic populations [11]. Previous research has demonstrated that sport-specific movement patterns and baseline neuromuscular characteristics can significantly influence intervention outcomes [12]. However, the comparative effectiveness across these distinct populations remains unclear.

While several systematic reviews have examined neuromuscular training effects on ACL injury incidence [13,14], limited synthesis exists specifically examining vGRF as a mechanistic outcome measure. Previous meta-analyses have either focused exclusively on injury rates [15] or examined broader biomechanical outcomes without specific attention to vGRF [16]. Furthermore, no systematic review has comprehensively evaluated the differential effects of movement retraining interventions between pivot-sport athletes and general athletic populations. Specifically, prior syntheses pooled mixed athletic populations without stratifying by sport demand and did not report a pivot-sport versus general-athletic contrast for peak landing force, which positions the present subgroup comparison as exploratory rather than confirmatory.

Within this context of substantial clinical and public health burden, primary prevention is itself a form of clinical care. Because peak vGRF during landing is a modifiable, biomechanically plausible risk marker that clinicians can target before injury occurs, synthesising the effect of movement retraining on this surrogate outcome can help inform the design of scalable, exercise-based ACL injury prevention programmes delivered in clinical and community or athletic health settings; because injury incidence is not measured by this surrogate, however, any downstream preventive effect remains to be confirmed in trials with clinical endpoints. Therefore, the aim of this study was to systematically review and meta-analyse the effectiveness of movement retraining interventions on vGRF in athletic populations. We frame this review as a mechanistic, surrogate-marker evidence synthesis intended to inform the design and prioritisation of population-level, exercise-based ACL injury prevention programmes, rather than as a demonstration of injury prevention per se. The primary outcome was the effect of movement retraining on peak vGRF during landing tasks. Secondary objectives were to compare effects between pivot-sport athletes and general athletic populations through subgroup analysis, and to examine the effect on knee flexion angle at initial contact as a candidate mechanistic outcome.

## 2. Materials and Methods

### 2.1. Study Design and Registration

This systematic review and meta-analysis was conducted in accordance with the Preferred Reporting Items for Systematic Reviews and Meta-Analyses (PRISMA) 2020 statement [17] (the completed PRISMA 2020 checklist is provided in Table S1) and the Cochrane Handbook for Systematic Reviews of Interventions, version 6.4 [18]. The protocol was prospectively registered in the International Prospective Register of Systematic Reviews (PROSPERO; CRD42025116119, registered 4 October 2025; https://www.crd.york.ac.uk/PROSPERO/view/CRD42025116119, accessed on 25 May 2026). The review was conducted in accordance with the registered protocol, with the following minor deviations or clarifications, reported transparently herein: (i) the literature search was completed on 25 May 2026 (the registered end date of 26 October 2025 was a planned timeline placeholder; the final search was extended to maximise comprehensiveness); (ii) the outcome construct of peak vGRF during landing was applied as a marker of impact attenuation associated with ACL injury risk modification—studies in which the intervention and outcome were framed within an alternative stretch-shortening-cycle (SSC) adaptation construct were considered outside the scope of this review. Because this outcome-construct refinement was specified before data extraction and applied uniformly, study selection rested on a pre-defined outcome rather than on observed results; nonetheless, any post-registration change to an outcome carries a risk of outcome-selection bias, and it accounts for the nine full-text exclusions on this ground. We flag this explicitly as a limitation. (iii) The final author list reflects ICMJE authorship criteria.

### 2.2. Eligibility Criteria

Studies were included based on the following PICOS criteria: (P) Athletes participating in organised sports, including pivot-sport athletes (basketball, volleyball, soccer, netball) and general athletic populations, without restrictions on age, sex, or competitive level; (I) Movement retraining interventions of any duration (1–8 weeks observed) designed to modify landing biomechanics, encompassing plyometric training (unilateral or bilateral), neuromuscular training, landing technique instruction with real-time or traditional feedback, or balance/proprioceptive training, framed within an impact-attenuation construct (i.e., intended to reduce landing forces as a marker of ACL injury risk); (C) Control conditions including no intervention, usual care, active control (e.g., standard warm-up or regular sport-specific training), or sham training; (O) Peak vertical ground reaction force (vGRF) measured during landing tasks using force platforms; (S) Randomised controlled trials (parallel-group or cluster designs). Studies in which both the intervention and the outcome were framed within an alternative mechanistic construct—such as enhancing stretch-shortening-cycle (SSC) force production or braking-phase force tolerance, where an increase in peak vGRF is expected and interpreted as evidence of beneficial adaptation—were considered outside the scope of this review and excluded. Operationally, a study was excluded on this ground only when both the intervention rationale and the stated outcome interpretation treated an increase in peak vGRF as the desired adaptation (for example, plyometric protocols aimed at enhancing stretch-shortening-cycle force production, or braking-phase force-tolerance training); studies reporting a reduction in peak vGRF as the intended impact-attenuation outcome were retained.

### 2.3. Search Strategy and Study Selection

A comprehensive systematic search was conducted from database inception through to 25 May 2026, without language restrictions. Electronic databases searched included MEDLINE via PubMed, Embase via Elsevier, and the Cochrane Central Register of Controlled Trials (CENTRAL). The search strategy combined controlled vocabulary (MeSH terms in PubMed; Emtree terms in Embase) with free-text terms in title, abstract, and keyword fields, structured around three concept blocks (Population, Intervention, Outcome) combined with the Boolean operator AND. Reference lists of relevant systematic reviews and the included studies were hand-screened for additional eligible studies. CENTRAL records were predominantly sourced from PubMed, Embase, or ClinicalTrials.gov registrations and therefore overlapped substantially with the other database searches. The full search strategies for each database are provided in the Supplementary Materials (Table S2).

The search strategy was developed using a combination of controlled vocabulary and free-text keywords. Population terms combined MeSH/Emtree headings for athletes and pivot sports with free-text terms (athlete*, player*, soccer, football, basketball, volleyball, netball, “pivot sport*”); intervention terms combined headings for plyometric/resistance/exercise therapy with free-text terms (“movement retraining”, “landing technique*”, “neuromuscular training”, “jump training”, plyometric*); and outcome terms combined biomechanics headings with free-text terms (“ground reaction force*”, vGRF, “landing force*”, “impact force*”, “loading rate*”). Concept blocks were combined with the AND operator. The complete, line-by-line search strategy for each database is provided in the Supplementary Materials (Table S2).

Reference lists of included studies and relevant systematic reviews were manually screened for additional eligible studies. All identified records were imported into reference management software for duplicate removal and screening.

Two reviewers (Y.H. and YH.K.) independently screened titles and abstracts and then assessed full texts against the eligibility criteria. Disagreements were resolved through discussion or, when necessary, consultation with a third reviewer (T.S.).

### 2.4. Data Extraction

A standardised data extraction form was developed and piloted on three randomly selected studies. Two reviewers (Y.H. and YH.K.) independently extracted: study characteristics (author, year, country, design); participant demographics (sample size, age [mean ± SD], sex distribution, sport type, competitive level); intervention characteristics (type, duration, frequency, intensity, progression, supervision); comparator conditions; outcome assessment methods (force-platform make/model, sampling frequency [range 500–1200 Hz], landing-task protocol); and outcome data (means, standard deviations, and sample sizes for vGRF at baseline and immediately post-intervention).

For studies reporting multiple time points, immediate post-intervention data were prioritised. When vGRF was reported in different units, values were converted to %BW for consistency. For correlation between pre- and post-test scores, we used r = 0.5 as recommended by the Cochrane Handbook when not reported [18]. Authors were contacted via email to obtain missing or unclear data.

### 2.5. Risk of Bias Assessment

**Figure 1:**
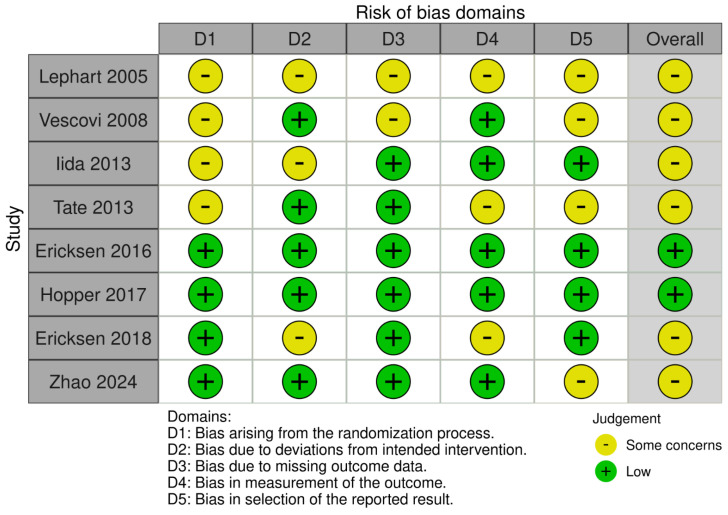
Risk of bias summary across included studies, assessed using the Cochrane Risk of Bias tool for randomised trials (RoB 2) [21,22,23,24,25,26,27,28]. Green = low risk; yellow = some concerns. No included study was rated at high risk of bias (red), so no red markers appear in the figure. D1 = randomisation process; D2 = deviations from intended interventions; D3 = missing outcome data; D4 = measurement of the outcome; D5 = selection of the reported result. Zhao 2024 [28] contributed two comparisons (2024a, unilateral plyometric jump training; 2024b, bilateral plyometric jump training) sharing a common control arm and an identical trial design; because RoB 2 is assessed at the trial level, the two comparisons receive a single risk-of-bias judgement and are shown as one row.

Risk of bias was independently assessed by two reviewers (Y.H. and YH.K.) using the revised Cochrane Risk of Bias tool (RoB 2) for randomised trials [19]. Five domains were evaluated (Figure 1): (D1) bias arising from the randomization process; (D2) bias due to deviations from intended interventions; (D3) bias due to missing outcome data; (D4) bias in measurement of the outcome; (D5) bias in selection of the reported result. Each domain was judged as “low risk”, “some concerns”, or “high risk”, leading to an overall risk-of-bias judgement. Disagreements were resolved through consensus discussion. Visual summaries were generated using the robvis web application [20].

### 2.6. Statistical Analysis

Meta-analyses were performed using Review Manager (RevMan) version 5.4.1 (The Cochrane Collaboration, Copenhagen, Denmark) [29] and Comprehensive Meta-Analysis (CMA) version 3.0 (Biostat, Englewood, NJ, USA) [30] for sensitivity analyses. For continuous outcomes, we calculated standardised mean differences (SMD) with 95% confidence intervals using post-intervention values. The direction of effect was coded such that negative SMD values indicated reduced vGRF (the direction hypothesised to be favourable for impact attenuation). Additional robustness analyses (variance-estimator, influence, and correlation-imputation sensitivity analyses described below) were performed in R version 4.6.0 using the metafor package.

Random-effects models (DerSimonian–Laird method) were used for all meta-analyses to account for expected heterogeneity across studies due to differences in populations, interventions, and assessment methods. Statistical heterogeneity was assessed using Cochran’s Q test and the I^2^ statistic, interpreted as: <30% (low), 30–60% (moderate), and >60% (substantial) heterogeneity. Subgroup analyses were performed based on athletic population type (pivot sports vs. general athletic) using mixed-effects models with Q tests for subgroup differences.

Publication bias was planned to be assessed using funnel plots and Egger’s test only when ≥10 studies were available [31]; because fewer than 10 studies were included (eight publications, nine comparisons), formal assessment of publication bias was therefore not undertaken. All statistical tests were two-tailed with significance set at *p* < 0.05. Hedges’ g was used as the effect-size metric, incorporating the small-sample correction factor J = 1 − 3/(4N − 9), where N = n_1_ + n_2_. Effect sizes were interpreted according to Cohen’s conventions: small (g ≈ 0.2), medium (g ≈ 0.5), and large (g ≥ 0.8).

To assess the robustness of the primary peak vGRF estimate, three additional analyses were conducted. First, because a pre-post correlation of r = 0.5 was assumed, following the Cochrane Handbook, only for the comparisons whose effect size was derived from change scores, we re-derived the change-score variances and re-pooled the effect under r = 0.3 and r = 0.7. Second, because the DerSimonian–Laird estimator can underestimate uncertainty when the number of studies is small, we re-estimated the pooled effect using restricted maximum likelihood (REML) for the between-study variance with a Hartung-Knapp-Sidik-Jonkman adjustment to the confidence interval. Third, we examined influence and outliers using standardised residuals, Cook’s distance, and DFFITS, re-pooling after removing any flagged comparison.

### 2.7. Certainty of Evidence Assessment

The Grading of Recommendations Assessment, Development, and Evaluation (GRADE) approach was used to rate the certainty of evidence for each outcome [32] (the full GRADE evidence profile is provided in Table S3). Two reviewers (Y.H. and YH.K.) independently rated certainty, starting at high certainty for randomised trials and downgrading for: risk of bias (if a majority of contributing studies had overall “some concerns” or “high risk”); inconsistency (substantial unexplained statistical heterogeneity, I^2^ > 60%); indirectness; imprecision (wide confidence intervals or failure to meet the optimal information size); and publication bias. Disagreements were resolved by discussion.

## 3. Results

### 3.1. Study Selection

**Figure 2:**
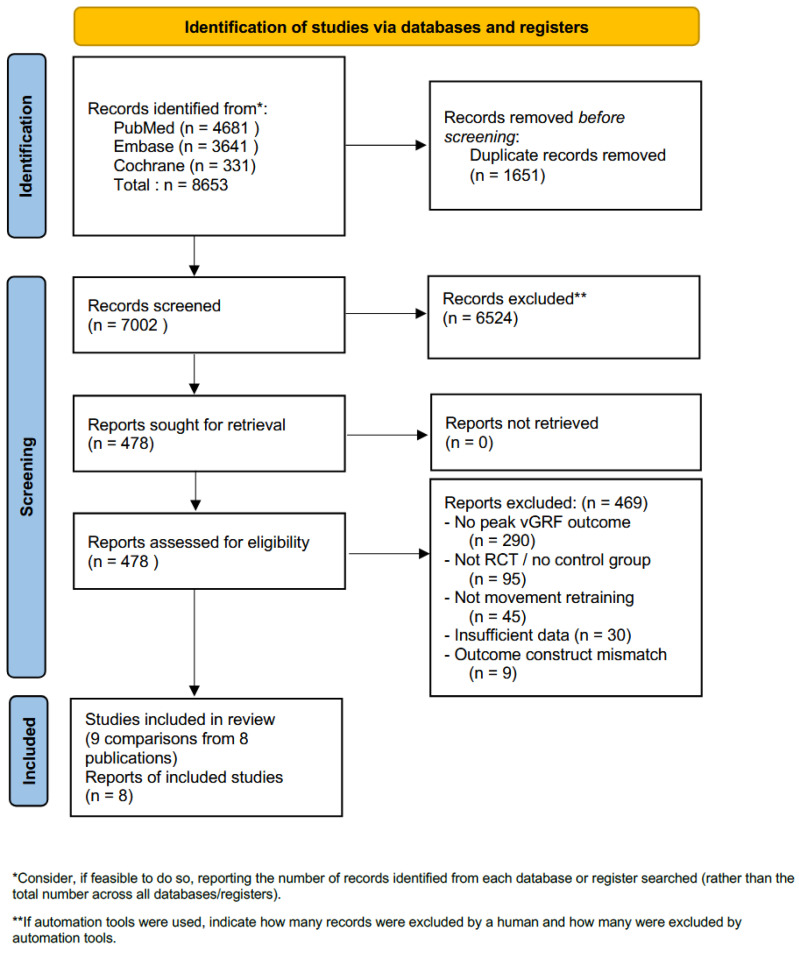
PRISMA 2020 flow diagram of study identification, screening, and inclusion.

The systematic literature search yielded 8653 records across the three databases (PubMed, *n* = 4681; Embase, *n* = 3641; Cochrane CENTRAL, *n* = 331). After removing 1651 duplicate records (1320 between PubMed and Embase identified by DOI and title matching, and 331 CENTRAL records that overlapped with the other two databases, as expected given CENTRAL’s aggregator structure, which is populated largely from MEDLINE, Embase, and trial registries; no CENTRAL record contributed a uniquely eligible study), 7002 unique records underwent title and abstract screening. Of these, 6524 were excluded as clearly ineligible. The remaining 478 reports were retrieved for full-text assessment, of which 469 were excluded with reasons recorded against the PICOS criteria (Figure 2). The principal reasons for full-text exclusion were: absence of peak vGRF measured during landing as a pre-specified outcome (*n* = 290); lack of a randomised design or of an adequate control group (*n* = 95); an intervention not consistent with the movement retraining construct (*n* = 45); insufficient data for meta-analysis (*n* = 30); and outcome construct mismatch (interventions framed within a stretch-shortening-cycle adaptation construct expecting an increase in peak vGRF rather than impact attenuation; *n* = 9). Nine comparisons, reported across eight publications (one publication contributing two independent comparisons), met all eligibility criteria and were included in the synthesis.

### 3.2. Study Characteristics

The eight included publications (nine comparisons) comprised 292 participants at the comparison level (intervention, *n* = 157; control, *n* = 135), with per-comparison sample sizes ranging from 18 to 50 participants (Table 1). Six of the eight publications (75%) exclusively recruited female participants (Lephart 2005 [21]; Vescovi 2008 [22]; Tate 2013 [24]; Hopper 2017 [26]; Ericksen 2016 [25]; Ericksen 2018 [27]), and two (25%) recruited only male participants (Zhao 2024 [28], youth basketball players; Iida 2013 [23], healthy adult men). Participant ages ranged from 12.2 to 25.3 years. As detailed in Table 1, one publication (Zhao 2024 [28]) contributed two comparisons sharing a common control arm; participant counts are therefore reported at the comparison level.

Intervention duration varied from 1 to 8 weeks, with training frequency ranging from 2 to 3 sessions per week. Movement retraining interventions included: plyometric training programmes (Lephart 2005 [21], Vescovi 2008 [22], Zhao 2024 [28]), neuromuscular training combining plyometric and strength exercises (Hopper 2017 [26]), jump-landing feedback training with real-time or traditional feedback methods (Ericksen 2016 [25], 2018 [27]), and landing technique instruction focusing on impact absorption (Iida 2013 [23], Tate 2013 [24]). Control conditions consisted primarily of regular sport-specific training or no additional intervention beyond habitual activities.

All studies assessed vGRF using force platforms, with sampling frequencies ranging from 500 to 1200 Hz (one study, Iida 2013 [23], collected force data at 1000 Hz and downsampled to 200 Hz for analysis; one study, Zhao 2024 [28], did not report the sampling rate). Landing tasks varied across studies and included drop jumps from 30 cm boxes, countermovement jumps, rebound jump-landings, and sport-specific landing manoeuvres.

### 3.3. Risk of Bias Results

Risk-of-bias assessment using the RoB 2 tool revealed variable methodological quality across the included studies (Figure 1). Two of the eight publications (25%) were judged at low risk of bias across all domains (Ericksen 2016 [25]; Hopper 2017 [26]). The remaining six publications (75%) showed some concerns in at least one domain, particularly the randomisation process (Domain 1) and selection of the reported result (Domain 5). Blinding of participants and personnel was not feasible given the nature of movement retraining interventions; however, the objective nature of force-platform measurement partially mitigates this concern. Outcome data were largely complete with minimal attrition (Domain 3). Some concerns raised under Domain 5 in several trials reflected limited prospective protocol availability and the post-registration refinement of the outcome construct rather than demonstrated selective reporting of results; we therefore did not conclude that selective reporting was absent.

### 3.4. Effects on Vertical Ground Reaction Forces

#### 3.4.1. Primary Outcome

**Figure 3:**
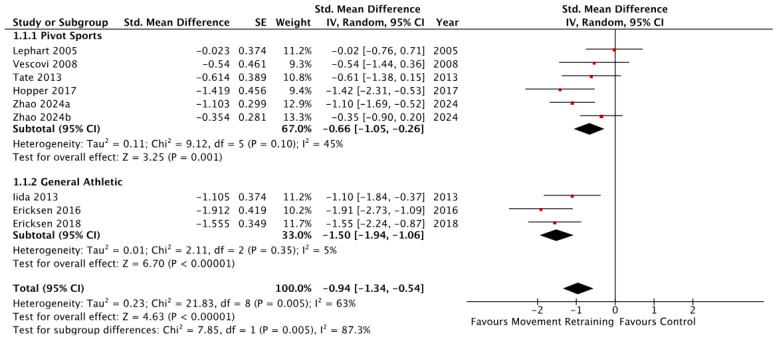
Forest plot of movement retraining effects on vertical ground reaction force (vGRF) during landing [21,22,23,24,25,26,27,28]. Random-effects meta-analysis (DerSimonian–Laird method) with Hedges’ g and 95% confidence intervals. Subgroup analysis by athletic population: pivot-sport athletes vs. general athletic populations. The standardised mean differences are reported as Hedges’ g (with small-sample correction); the column heading generated by the analysis software (“Std. Mean Difference”) refers to the same quantity. Squares represent individual study effect estimates (size proportional to study weight); horizontal lines indicate 95% confidence intervals; diamonds represent the pooled random-effects estimate.

Movement retraining significantly reduced peak vGRF during landing compared with control conditions (Hedges’ g = −0.94, 95% CI −1.34 to −0.54; Z = 4.63, *p* < 0.00001; 9 comparisons across 8 publications, 292 participants; Figure 3). This represents a large effect on the biomechanical marker; however, given the substantial heterogeneity (I^2^ = 63%) and the low certainty of evidence (GRADE), the magnitude should be regarded as imprecise and interpreted with caution. Statistical heterogeneity was substantial (I^2^ = 63%; Tau^2^ = 0.23; Cochran’s Q = 21.83, df = 8, *p* = 0.005), and a random-effects model was therefore used. Because vGRF is a surrogate biomechanical marker rather than a clinical injury endpoint, this effect indicates a favourable change in landing mechanics rather than a demonstrated reduction in ACL injury incidence. Notably, the largest single effect (Ericksen 2016 [25], g = −1.91) arose from one of the smaller analysable samples (*n* = 32), a pattern consistent with small-study effects; because fewer than 10 comparisons were available, funnel-plot assessment of this possibility was not feasible, so some overestimation of the pooled effect cannot be excluded.

#### 3.4.2. Subgroup Analysis by Athletic Population

Subgroup analysis revealed differential effects by athletic population type (test for subgroup differences: Chi^2^ = 7.85, df = 1, *p* = 0.005, I^2^ = 87.3%).

Pivot-sport athletes: Across comparisons involving athletes participating in cutting and pivoting sports—basketball, volleyball, soccer, and netball, including mixed pivot-sport and high-school pivot-sport cohorts (6 comparisons, 194 participants), movement retraining produced a moderate reduction in peak vGRF (Hedges’ g = −0.66, 95% CI −1.05 to −0.26; Z = 3.25, *p* = 0.001). Within-subgroup heterogeneity was moderate (I^2^ = 45%; Q = 9.12, df = 5, *p* = 0.10).

General athletic populations: Comparisons involving healthy adults or recreational athletes not engaged in cutting/pivoting sports (3 comparisons, 98 participants) demonstrated a larger effect (Hedges’ g = −1.50, 95% CI −1.94 to −1.06; Z = 6.70, *p* < 0.00001), with minimal heterogeneity (I^2^ = 5%; Q = 2.11, df = 2, *p* = 0.35), indicating consistent effects across these studies.

### 3.5. Secondary Outcome: Knee Flexion Angle

**Figure 4:**
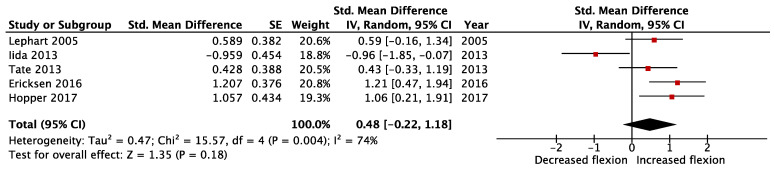
Forest plot of movement retraining effects on knee flexion angle at initial contact during landing [21,23,24,25,26]. Random-effects meta-analysis with Hedges’ g and 95% confidence intervals. The standardised mean differences are reported as Hedges’ g; on the horizontal axis, positive values (right) indicate increased knee flexion at initial contact. Squares represent individual study effect estimates (size proportional to study weight); horizontal lines indicate 95% confidence intervals; the diamond represents the pooled random-effects estimate.

Knee flexion angle at initial contact was analysed in five studies reporting this biomechanical outcome (Figure 4). Movement retraining showed a non-significant trend toward increased knee flexion angle at initial contact (Hedges’ g = 0.48, 95% CI −0.22 to 1.18; Z = 1.35, *p* = 0.18; 5 studies). The point estimate is in the direction of greater knee flexion (a more flexed landing posture), which would be biomechanically favourable for force attenuation if confirmed in adequately powered studies; however, the confidence interval crossed the null and the result was not statistically significant. Heterogeneity was substantial (I^2^ = 74%; Tau^2^ = 0.47; Q = 15.57, df = 4, *p* = 0.004). Given the non-significant pooled estimate, the confidence interval crossing the null, and the high heterogeneity (I^2^ = 74%), no mechanistic inference should be drawn from this secondary outcome.

### 3.6. Sensitivity Analyses

Leave-one-out sensitivity analysis demonstrated robust results, with the pooled estimate remaining statistically significant regardless of which single comparison was removed (Hedges’ g range −0.82 to −1.07). Restricting the analysis to the two publications judged at low risk of bias yielded a comparable estimate (Hedges’ g = −1.18, 95% CI −1.67 to −0.69), albeit with reduced precision owing to the smaller sample.

The pooled effect was robust to the assumed pre-post correlation: re-deriving change-score variances under r = 0.3 and r = 0.7 yielded pooled estimates of Hedges’ g = −0.90 (95% CI −1.27 to −0.52) and g = −1.03 (95% CI −1.53 to −0.54), respectively, compared with g = −0.94 (95% CI −1.34 to −0.54) at r = 0.5, with the direction and statistical significance unchanged (Table S4). Re-estimation using REML with a Hartung-Knapp-Sidik-Jonkman adjustment gave g = −0.94 (95% CI −1.41 to −0.47; *p* = 0.002), essentially unchanged in magnitude from the primary DerSimonian–Laird estimate but with a wider, more conservative interval, and the effect remained statistically significant. Influence diagnostics (standardised residuals, Cook’s distance, and DFFITS) flagged no comparison as influential; the largest residual (Ericksen 2016 [25]) did not exceed the conventional threshold, and removing that comparison, which had the largest effect (g = −1.91), produced a pooled estimate of g = −0.83 (95% CI −1.21 to −0.45), consistent with the leave-one-out range and the primary result.

### 3.7. Publication Bias

Because fewer than 10 studies were available (8 publications, 9 comparisons), funnel-plot asymmetry and Egger’s test were not assessed, in line with Cochrane recommendations [31]; the possibility of small-study effects therefore cannot be excluded.

### 3.8. Certainty of Evidence

Using the GRADE approach, the certainty of evidence for the primary outcome (vGRF reduction, all populations) was rated low, downgraded for risk of bias (some concerns in 6 of 8 publications) and for inconsistency (substantial heterogeneity, I^2^ = 63%). The certainty for the pivot-sport subgroup was also rated low, downgraded for risk of bias (some concerns in 4 of 6 comparisons) and for imprecision (the subgroup did not meet the optimal information size); it was not downgraded for inconsistency because within-subgroup heterogeneity was only moderate (I^2^ = 45%, below the I^2^ > 60% threshold) (Table 2).

## 4. Discussion

Anterior cruciate ligament (ACL) injury imposes a substantial burden on patients, clinicians, and health systems: it is common in young and recreationally active people and is rarely an isolated event, commonly entailing reconstructive surgery, a prolonged and resource-intensive rehabilitation course, lost participation, and an elevated long-term risk of post-traumatic knee osteoarthritis. This systematic review and meta-analysis evaluated the effect of movement retraining on peak vertical ground reaction force (vGRF), a surrogate biomechanical marker, during landing. On the basis of low-certainty evidence with substantial heterogeneity (I^2^ = 63%), the pooled estimate was Hedges’ g = −0.94 (95% CI −1.34 to −0.54). Although large by Cohen’s conventions, this estimate is imprecise, and because vGRF is a surrogate for rather than a measure of ACL injury, it represents a favourable change in a biomechanical risk marker rather than demonstrated injury prevention. The result extends prior injury-prevention research by focusing on a mechanistic outcome, suggesting that diverse training approaches—plyometric training, neuromuscular programmes, and feedback-based interventions—can modify this landing force marker. Exercise-based prevention programmes are generally regarded as relatively low-cost and potentially scalable within existing training and physical education settings; optimising and continuing to evaluate programmes that modify such risk markers is therefore a clinically and public-health-relevant strategy, even though confirmation of reduced injury incidence will require trials with clinical endpoints.

These findings carry epidemiological context. With more than 250,000 ACL reconstructions performed annually in the United States, approximately 70% of which occur through non-contact mechanisms during landing and cutting, interventions that favourably modify landing mechanics are of interest for prevention programmes; the burden, however, motivates prevention without itself establishing that the observed change in this surrogate marker reduces injury incidence—although, because injury incidence was not measured here, any downstream effect on injury rates remains to be demonstrated in trials with clinical endpoints. The use of Hedges’ g provides bias-corrected estimates that are particularly important given the small-to-moderate samples (18–50 participants) typical of biomechanical intervention studies.

A differential response was observed between pivot-sport athletes (Hedges’ g = −0.66) and general athletic populations (Hedges’ g = −1.50). The subgroup test yielded *p* = 0.005; however, with only three general-athletic comparisons (98 participants), this contrast is exploratory and may partly reflect sampling variability. If confirmed in adequately powered trials, the larger apparent effect in general populations may reflect greater adaptability of motor patterns in individuals without extensive sport-specific movement experience, possibly involving improved proprioceptive integration and muscle pre-activation timing [33,34]. It contrasts with the assumption that highly trained athletes would respond more strongly, and is consistent with a possible ceiling effect in those with established movement patterns [35].

Although smaller than in general populations, the moderate effect in pivot-sport athletes (g = −0.66) remains potentially relevant, given that prospective data link higher pre-injury landing forces (approximately 20% greater) to subsequent ACL injury [36]. Even modest reductions in landing forces, accumulated across the many landing cycles of training and competition, may be biomechanically meaningful, though their translation to injury risk requires confirmation in studies with clinical endpoints.

A secondary analysis examined knee flexion angle at initial contact (Hedges’ g = 0.48, 95% CI −0.22 to 1.18; *p* = 0.18). The point estimate was in the direction of greater knee flexion—a more flexed landing posture that would, in principle, reduce peak forces by increasing the time and distance over which deceleration occurs through the impulse–momentum relationship [37]. However, the effect was not statistically significant and the confidence interval crossed the null. Because the pooled estimate was not statistically significant, the confidence interval crossed the null, and heterogeneity was substantial (I^2^ = 74%), no reliable conclusion about sagittal-plane landing kinematics can be drawn, and the result should not be taken to support a specific mechanistic pathway. Adequately powered studies measuring both knee flexion and peak vGRF are needed.

That diverse intervention types (plyometric, neuromuscular, and feedback-based) produced broadly similar reductions in landing force suggests that more than one training pathway may be effective, although the present data do not allow these pathways to be distinguished.

The substantial heterogeneity (I^2^ = 63%) likely reflects methodological and population diversity across interventions. Programmes emphasising real-time feedback may operate through explicit motor-learning pathways [38], whereas traditional plyometric training may target implicit neuromuscular adaptations [39]. That diverse approaches achieved broadly similar directional outcomes suggests multiple viable pathways, allowing prevention programme design to be adapted to the resources of physical education, primary care, and community health settings. The relatively short intervention durations (1–8 weeks) indicate that measurable adaptations can occur within the brief programme windows feasible in school, community, and clinical prevention settings [40].

Beyond statistical heterogeneity, the included trials differed substantially in clinical and methodological characteristics: populations ranged from young basketball and netball players to recreational athletes and healthy adults; interventions spanned plyometric and jump training, real-time and video feedback, broader neuromuscular programmes, and explicit landing instruction; and landing tasks varied from standardised drop jumps to sport-specific manoeuvres. This clinical diversity limits the extent to which a single pooled estimate can be attributed to any one intervention component, and means the pooled value should be read as an average across heterogeneous programmes. The random-effects model and the pre-specified sport-demand subgroup analysis address this only in part, and residual intervention heterogeneity contributed to the GRADE downgrade for inconsistency.

Subgroup analysis revealed important patterns in heterogeneity distribution. Within pivot sports, moderate heterogeneity (I^2^ = 45%) suggests relatively consistent responses despite differences in specific sports (basketball, volleyball, soccer, netball). In contrast, general athletic populations showed minimal heterogeneity (I^2^ = 5%), indicating highly consistent intervention effects in non-specialised athletes. This pattern has implications for prevention programme delivery: standardised protocols may be sufficient in general and recreational populations, whereas the higher-risk pivot-sport population may require more intensive, longer, or sport-specific protocols.

These findings do not establish that movement retraining prevents ACL injury. They indicate that several exercise interventions can reduce a biomechanical marker that has been associated, in prospective screening studies, with injury risk [41]. Whether modifying this marker lowers injury incidence remains unknown. Pending confirmation in adequately powered trials with clinical endpoints, the results provide a rationale for designing and testing prevention programmes rather than a basis for implementing them.

Because the outcome is a continuous surrogate biomechanical marker (peak vGRF) rather than a binary clinical event, event-based clinical metrics are not applicable, and the standardised mean differences reported here should not be converted into injury incidence estimates. The clinical value of the observed force reductions ultimately depends on confirmation in trials that measure ACL injury incidence directly.

Several implementation observations emerge descriptively from the included studies. Feedback-based programmes (real-time or video) showed a non-significant tendency toward larger effects than approaches without feedback, although all intervention types produced reductions in landing force. Session frequency ranged from daily to twice weekly; a formal dose–response analysis was not undertaken given the small number of comparisons. The shortest intervention (one week) still produced a moderate effect, whereas longer programmes (6–8 weeks) tended to yield larger effects, suggesting flexibility in scheduling, though these patterns are observational and should be interpreted cautiously.

### Limitations

Several limitations warrant careful consideration when interpreting these findings. While Hedges’ g provides appropriate bias correction for small samples, the varying study sizes and methodological approaches introduce uncertainty that pooled estimates cannot fully eliminate. The predominance of female participants (approximately 75% across studies) limits generalizability to male athletes, particularly given established sex differences in landing biomechanics, neuromuscular control patterns, and ACL injury mechanisms. Females typically demonstrate greater dynamic knee valgus, reduced hip and knee flexion angles, altered trunk control, and different muscle activation patterns during landing—factors that may substantially influence intervention responsiveness and optimal training approaches. Importantly, no included trial reported sex-disaggregated peak vGRF data sufficient for a sex-specific pooled analysis; we were therefore unable to test whether the effect differs by sex, and as a result, sex-stratified reporting should be a priority for future trials. The direction of any resulting bias is uncertain: because female athletes typically display higher pre-landing valgus and vGRF values, they may have greater scope for force reduction (inflating the apparent effect) or may approach a biomechanical floor that constrains it; the effect in male athletes therefore cannot be inferred from these data.

Follow-up periods were short across the included studies, precluding assessment of the retention of effects, which is crucial for understanding long-term prevention potential. While immediate post-intervention improvements are encouraging, the durability of neuromuscular adaptations is unknown, and maintenance or booster training may be required to preserve any benefit across a competitive season.

Laboratory-based assessments, while providing controlled measurement conditions, may not fully capture sport-specific scenarios where multiple factors influence movement patterns. Fatigue accumulation during competition, divided attention from cognitive demands, reactive responses to opponents, and environmental constraints (surface variations, weather conditions) all potentially modify landing mechanics in ways not reflected in controlled testing. This ecological validity concern suggests our effect sizes may overestimate real-world intervention benefits, though the consistency of findings across varied testing protocols provides some reassurance.

The substantial heterogeneity, only partially explained by the population subgroups, indicates additional unmeasured moderators of effectiveness. Training volume, degree of supervision (from fully supervised to unsupervised home programmes), and baseline movement competency varied across studies and were inconsistently reported, precluding systematic evaluation. Future primary studies should standardise the reporting of these potential moderators.

The inability to conduct robust publication bias assessment due to the limited number of studies (fewer than 10; 8 publications, 9 comparisons) raises concerns about potential effect size overestimation. Small-study effects, where smaller studies show larger effect sizes, could inflate our pooled estimates. While our use of Hedges’ g provides some protection against small-sample bias, and sensitivity analyses showed consistent effects after excluding smaller studies, the possibility of selective bias favouring positive results cannot be excluded.

Methodological features of the included studies affect the certainty of evidence. Participant and personnel blinding is not feasible in movement-training interventions, introducing potential performance bias, although objective force-platform measurement partially mitigates this concern. Force platforms varied in specification (sampling frequencies of 500–1200 Hz, with one study downsampling to 200 Hz for analysis and one not reporting the rate; differing manufacturers and models), potentially contributing to the observed heterogeneity. Variability in landing tasks—from standardised drop jumps to sport-specific manoeuvres—reflects real-world diversity but complicates direct comparison of effects.

## 5. Conclusions

This meta-analysis indicates that movement retraining interventions produce a large reduction in peak vertical ground reaction force during landing (Hedges’ g = −0.94), a biomechanical risk marker associated with ACL injury, with low certainty of evidence (GRADE). This estimate rests on only eight publications (9 comparisons, 292 participants), so the statistically significant pooled effect should be regarded as preliminary. Effects appeared larger in general athletic populations than in pivot-sport athletes, suggesting that programme design may need to be tailored to the target population. A non-significant trend toward increased knee flexion angle provides a plausible but unconfirmed mechanistic pathway. Because the synthesis is based on a surrogate biomechanical outcome rather than injury incidence, and because the certainty of evidence is low owing to risk of bias and heterogeneity, these findings should be regarded as supporting the potential value of movement retraining for modifying landing mechanics rather than as evidence of proven injury prevention. Adequately powered randomised trials with clinical (injury-incidence) endpoints, longer follow-up, and sex-stratified analyses are needed to determine whether these biomechanical changes translate into reduced ACL injury rates. As a surrogate marker evidence synthesis, this review is intended to inform the design and prioritisation of population-level ACL injury prevention programmes, while making explicit that a corresponding reduction in injury incidence remains to be confirmed.

## Figures and Tables

**Table 1:** Characteristics of included studies.

Study	Design	*n*	Population (Sex)	Intervention	Comparator	Duration	Force Plate (Hz)	Hedges’ g [95% CI]
Lephart 2005 [21]	RCT	27 (14/13)	High-school pivot-sport athletes (F)	Plyometric training	Basic resistance training	8 wk	Bertec (1200)	−0.02 [−0.76, 0.71]
Vescovi 2008 [22]	RCT	18 (10/8) a	College basketball (F)	Sportsmetrics plyometric	No additional training	6 wk	Kistler Quattro (500)	−0.54 [−1.44, 0.36]
Tate 2013 [24]	RCT	26 (13/13)	Recreational pivot-sport athletes (volleyball/basketball/soccer) (F)	Jump training instruction	Sham training	1 wk	AMTI (1200)	−0.61 [−1.38, 0.15]
Hopper 2017 [26]	RCT	23 (13/10)	Netball athletes (F)	Neuromuscular (plyo + strength)	Regular netball training	6 wk	Kistler 9287CA (1000)	−1.42 [−2.31, −0.53]
Zhao 2024a (uPJT) [28]	RCT	50 (25/25) b	Basketball (M, youth)	Unilateral plyometric (uPJT)	Regular basketball training	8 wk	ForceDecks (NR)	−1.10 [−1.69, −0.52]
Zhao 2024b (bPJT) [28]	RCT	50 (25/25) b	Basketball (M, youth)	Bilateral plyometric (bPJT)	Regular basketball training	8 wk	ForceDecks (NR)	−0.35 [−0.90, 0.20]
Iida 2013 [23]	RCT	20 (10/10)	Healthy adults (M)	Landing training (no jumping)	No training	2 wk	Kistler (1000) c	−1.10 [−1.84, −0.37]
Ericksen 2016 [25]	RCT	32 (16/16) d	Healthy adults (F)	Real-time + traditional feedback	No intervention	4 wk	AMTI OR6-5 (1000)	−1.91 [−2.73, −1.09]
Ericksen 2018 [27]	RCT	46 (31/15)	Recreational athletes (F)	Jump-landing feedback	No intervention	4 wk	AMTI OR6-5 (1000)	−1.55 [−2.24, −0.87]

*n* = Participants (intervention/control) enrolled and counted at the comparison level. (a) Vescovi 2008 [22] enrolled 20; the vGRF analysis included 18 (10 intervention, 8 control) after exclusion of 2 outliers. (b) Zhao 2024 [28] enrolled 75 unique participants and contributes two comparisons (uPJT, bPJT) that share one common control arm (*n* = 25); counts are therefore reported at the comparison level. (c) Iida 2013 [23]’s force data were collected at 1000 Hz and downsampled to 200 Hz for analysis. (d) Ericksen 2016 [25] enrolled 48 (three arms); 32 (16 intervention, 16 control) contributed analysable vGRF data. F = female; M = male; NR = not reported; RCT = randomised controlled trial; uPJT = unilateral plyometric jump training; bPJT = bilateral plyometric jump training; vGRF = vertical ground reaction force; Hz = hertz. Across the eight publications (nine comparisons), the total analysed sample was 292 participants (intervention *n* = 157; control *n* = 135).

**Table 2:** Summary of findings: movement retraining compared to control for reducing landing forces in athletic populations.

Summary of findings						
Movement Retraining Compared to Control in Athletic Populations						
Patient or Population: Athletic Populations						
Setting: Community, School/Physical Education, and Clinical Sports Medicine Prevention Settings						
Intervention: Movement Retraining						
Comparison: Control						
Outcome № of Participants (Studies)	Relative Effect (95% CI)	Anticipated Absolute Effects (95% CI)			Certainty	What Happens
		Control	Movement Retraining	Difference		
Landing forces (peak vGRF), all populations Assessed with: force platform (N/kg or %BW) No. of participants: 292 (8 publications, 9 comparisons)	-	-	-	SMD 0.94 SD lower (1.34 lower to 0.54 lower)	⨁⨁◯◯ Low a,b	Movement retraining may reduce peak vGRF during landing (Hedges’ g −0.94, 95% CI −1.34 to −0.54). As vGRF is a surrogate biomechanical marker, the effect on actual ACL injury incidence is uncertain.
Landing forces (peak vGRF), pivot-sport athletes Assessed with: force platform (N/kg or %BW) No. of participants: 194 (6 comparisons)	-	-	-	SMD 0.66 SD lower (1.05 lower to 0.26 lower)	⨁⨁◯◯ Low a,c,d	Movement retraining may reduce peak vGRF during landing in pivot-sport athletes (Hedges’ g −0.66, 95% CI −1.05 to −0.26).

Outcomes are continuous (standardised mean differences); relative-effect and absolute-risk columns are not applicable. GRADE Working Group grades of evidence. High certainty: we are very confident that the true effect lies close to that of the estimate of the effect. Moderate certainty: we are moderately confident in the effect estimate. Low certainty: our confidence in the effect estimate is limited. Very low certainty: we have very little confidence in the effect estimate. CI = confidence interval; SMD = standardised mean difference; vGRF = vertical ground reaction force. (a) Downgraded one level for risk of bias: 6 of 8 publications (75%) had some concerns on the RoB 2 tool, particularly with regard to the randomisation process (Domain 1) and selection of the reported result (Domain 5). (b) Downgraded one level for inconsistency: substantial statistical heterogeneity (I^2^ = 63%) across the pooled estimate. (c) Within the pivot-sport subgroup, 4 of 6 comparisons had some concerns on the RoB 2 tool. (d) Downgraded for imprecision: the subgroup sample size did not meet the optimal information size (OIS ≈ 400 participants). Shaded columns indicate the anticipated absolute effects (assumed and corresponding risks); shading is used for visual grouping only. GRADE certainty is shown by filled/open circles: ⊕⊕◯◯ Low.

## Data Availability

All data analysed in this systematic review and meta-analysis are derived from previously published studies, which are cited in the References. The full search strategies, the PRISMA 2020 checklist, and the GRADE evidence profile are provided as Supplementary Materials (Tables S1–S4); the per-study risk-of-bias (RoB 2) assessment is shown in Figure 1. The extracted data, screening log, and meta-analytic input files (Review Manager v5.4.1 and Comprehensive Meta-Analysis v3.0) are available from the corresponding author upon reasonable request.

## Supplementary Materials

The following supporting information can be downloaded at: https://www.mdpi.com/article/10.3390/jfmk11030259/s1; Table S1: Full search strategies for MEDLINE/PubMed, Embase, and Cochrane CENTRAL; Table S2: PRISMA 2020 checklist; Table S3: GRADE evidence profile for the primary and secondary outcomes; Table S4: Sensitivity of the pooled peak vGRF estimate to the assumed pre-post correlation (r = 0.3, 0.5, 0.7). The per-study risk-of-bias (RoB 2) assessment is presented in Figure 1.
