# Supplementary material for: Movement Retraining and Peak Landing Force, a Modifiable Anterior Cruciate Ligament Injury Risk Marker, in Athletes: A Systematic Review and Meta-Analysis for Primary Prevention
Source: J Funct Morphol Kinesiol. 2026 Jun 29;11(3):259. doi: 10.3390/jfmk11030259 (PMC13398292; doi:10.3390/jfmk11030259)
Supplement: Supplementary file 1 [file jfmk-11-00259-s001.zip › Table_S1_Search_Strategies.pdf]

Supplementary Material S1 — Full Search Strategies

Manuscript: Movement Retraining and Peak Landing Force, a Modifiable Anterior Cruciate Ligament Injury Risk Marker, in Athletes: A Systematic Review and Meta-Analysis for Primary Prevention

Final search date: 25 May 2026

Total records identified: 8,653 (PubMed 4,681; Embase 3,641; Cochrane CENTRAL 331)

S1.1 — MEDLINE via PubMed

Search executed: 25 May 2026

```
(("Athletes"[MeSH] OR "Athletic Performance"[MeSH] OR "Soccer"[MeSH] OR
"Basketball"[MeSH] OR "Volleyball"[MeSH]) OR (athlete*[tiab] OR player*[tiab] OR
soccer[tiab] OR football[tiab] OR basketball[tiab] OR volleyball[tiab] OR netball[tiab]
OR handball[tiab] OR "team sport*"[tiab] OR "pivot sport*"[tiab] OR "cutting
sport*"[tiab] OR "multidirectional sport*"[tiab]))
AND
(("Exercise Therapy"[MeSH] OR "Exercise"[MeSH] OR "Resistance Training"[MeSH] OR
"Physical Therapy Modalities"[MeSH]) OR ("movement retraining"[tiab] OR "movement re-
training"[tiab] OR "landing technique*"[tiab] OR "landing training"[tiab] OR "landing
retraining"[tiab] OR "jump-landing training"[tiab] OR "neuromuscular training"[tiab] OR
"jump training"[tiab] OR plyometric*[tiab] OR "biomechanical training"[tiab] OR
"technique training"[tiab] OR "injury prevention program*"[tiab] OR "training
program*"[tiab] OR "training intervention*"[tiab]))
AND
(("Biomechanical Phenomena"[MeSH] OR "Task Performance and Analysis"[MeSH]) OR ("landing
force*"[tiab] OR "ground reaction force*"[tiab] OR GRF[tiab] OR vGRF[tiab] OR "vertical
ground reaction force*"[tiab] OR "peak force*"[tiab] OR "peak vertical force*"[tiab] OR
"impact force*"[tiab] OR "landing kinetic*"[tiab] OR "landing biomechanic*"[tiab] OR
"loading rate*"[tiab] OR "landing mechanic*"[tiab]))
```

Records identified: 4,681

S1.2 — Embase via Elsevier

Search executed: 25 May 2026

Line	Query	Records
#1	'soccer'/exp OR 'basketball'/exp OR 'volleyball'/exp OR 'netball'/exp OR 'athlete'/exp	111,331
#2	soccer:ti,ab OR football:ti,ab OR basketball:ti,ab OR volleyball:ti,ab OR netball:ti,ab OR handball:ti,ab OR 'team sport*':ti,ab OR 'pivot sport*':ti,ab OR 'cutting sport*':ti,ab OR 'multidirectional sport*':ti,ab OR athlete*:ti,ab OR 'athletic population*':ti,ab	129,181
#3	#1 OR #2	155,675
#4	'plyometric training'/exp OR 'resistance training'/exp OR 'athletic performance'/exp OR 'sport injury'/exp	104,936
#5	'movement retraining':ti,ab OR 'movement re-training':ti,ab OR 'landing technique*':ti,ab OR 'landing training':ti,ab OR 'landing retraining':ti,ab OR 'jump-	107,143

	landing training':ti,ab OR 'neuromuscular training':ti,ab OR 'jump training':ti,ab OR plyometric*:ti,ab OR 'biomechanical training':ti,ab OR 'technique training':ti,ab OR 'injury prevention program*':ti,ab OR 'training program*':ti,ab OR 'training intervention*':ti,ab	
**#6**	#4 OR #5	202,578
**#7**	'ground reaction force'/exp OR 'biomechanics'/exp	164,915
**#8**	'landing force*':ti,ab OR 'ground reaction force*':ti,ab OR grf:ti,ab OR vgrf:ti,ab OR 'vertical ground reaction force*':ti,ab OR 'peak force*':ti,ab OR 'peak vertical force*':ti,ab OR 'impact force*':ti,ab OR 'landing kinetic*':ti,ab OR 'landing biomechanic*':ti,ab OR 'loading rate*':ti,ab OR 'landing mechanic*':ti,ab	28,859
**#9**	#7 OR #8	183,654
**#10**	#3 AND #6 AND #9	3,641

Records identified: 3,641

S1.3 — Cochrane Central Register of Controlled Trials (CENTRAL)

Search executed: 25 May 2026

Line	Query	Records
**#1**	[mh Athletes] OR [mh "Athletic Performance"] OR [mh Soccer] OR [mh Basketball] OR [mh Volleyball]	12,836
**#2**	(athlete* OR player* OR soccer OR football OR basketball OR volleyball OR netball OR handball OR "team sport*" OR pivot NEXT sport* OR cutting NEXT sport* OR multidirectional NEXT sport*):ti,ab,kw	17,854
**#3**	#1 OR #2	25,827
**#4**	[mh "Plyometric Exercise"] OR [mh "Resistance Training"] OR [mh "Exercise Therapy"]	24,903
**#5**	("movement retraining" OR "movement re-training" OR "landing technique*" OR "landing training" OR "landing retraining" OR "jump-landing training" OR "neuromuscular training" OR "jump training" OR plyometric* OR "biomechanical training" OR "technique training" OR "injury prevention program*" OR "training program*" OR "training intervention*"):ti,ab,kw	21,578
**#6**	#4 OR #5	43,318
**#7**	[mh "Biomechanical Phenomena"]	4,533
**#8**	("landing force*" OR "ground reaction force*" OR GRF OR vGRF OR "vertical ground reaction force*" OR "peak force*" OR "peak vertical force*" OR "impact force*" OR "landing kinetic*" OR "landing biomechanic*" OR "loading rate*" OR "landing mechanic*"):ti,ab,kw	1,560
**#9**	#7 OR #8	5,759
**#10**	#3 AND #6 AND #9	331

Records identified: 331

Note: CENTRAL records were predominantly sourced from PubMed, Embase, or ClinicalTrials.gov registrations, and therefore overlapped substantially with the other two database searches.

S1.4 — Total Records & Deduplication

Stage	n
**PubMed**	4,681
**Embase**	3,641
**Cochrane CENTRAL**	331
**Total identified**	**8,653**
**Duplicates removed (DOI + title matching across PubMed/Embase)**	1,320
**Duplicates removed (CENTRAL overlapping with PubMed/Embase)**	331
**Total duplicates removed**	**1,651**
**Unique records screened**	**7,002**

S1.5 — Hand-search

Reference lists of included studies and relevant systematic reviews/meta-analyses (Webster and Hewett, 2018; Huang et al., 2020; Taylor et al., 2015) were screened for additional eligible studies. No additional studies meeting eligibility were identified.

[End of Supplementary S1]
