# Supplementary material for: Movement Retraining and Peak Landing Force, a Modifiable Anterior Cruciate Ligament Injury Risk Marker, in Athletes: A Systematic Review and Meta-Analysis for Primary Prevention
Source: J Funct Morphol Kinesiol. 2026 Jun 29;11(3):259. doi: 10.3390/jfmk11030259 (PMC13398292; doi:10.3390/jfmk11030259)
Supplement: Supplementary file 1 [file jfmk-11-00259-s001.zip › Table_S2_PRISMA_2020_Checklist.pdf]

Supplementary Table S2 — PRISMA 2020 Checklist

Manuscript: Movement Retraining and Peak Landing Force, a Modifiable Anterior Cruciate Ligament Injury Risk Marker, in Athletes: A Systematic Review and Meta-Analysis for Primary Prevention

"Location reported" refers to the section of the main manuscript.

TITLE

#	Item	Reported (Location)
1	Identify the report as a systematic review.	Title — "A Systematic Review and Meta-Analysis"

ABSTRACT

#	Item	Reported (Location)
2	See the PRISMA 2020 for Abstracts checklist (structured abstract).	Abstract (Background/Objectives, Methods, Results, Conclusions)

INTRODUCTION

#	Item	Reported (Location)
3	Describe the rationale for the review in the context of existing knowledge.	§1 Introduction
4	Provide an explicit statement of the objective(s) or question(s).	§1 Introduction (final paragraph)

METHODS

#	Item	Reported (Location)
5	Specify the inclusion and exclusion criteria and how studies were grouped.	§2.2 Eligibility Criteria (PICOS)
6	Specify all databases, registers and other sources searched; date last searched.	§2.3 Search Strategy; Supplementary Table S1 (last searched 25 May 2026)
7	Present the full search strategies for all sources.	Supplementary Table S1
8	Specify the methods used to decide whether a study met the inclusion criteria (number of reviewers, independence, automation).	§2.3 (two independent reviewers, Y.-H.U. and Y.K.; third reviewer T.C. for disagreements)
9	Specify the methods to collect data (number of reviewers, independence).	§2.4 Data Extraction (two independent reviewers)
10a	List and define all outcomes for which data were sought.	§2.2 (O); §2.4 — primary: peak vGRF; secondary: knee flexion angle
10b	List and define all other variables for which data were sought.	§2.4 (study, participant, intervention characteristics)
11	Specify methods to assess risk of	§2.5 (RoB 2; two independent

	bias (tool, number of reviewers, independence).	reviewers H.J. and Y.-H.U.)
**12**	Specify the effect measure(s) used in the synthesis or presentation of results.	§2.6 (Hedges' g / standardised mean difference)
**13a**	Describe the processes used to decide which studies were eligible for synthesis.	§2.2, §2.6
**13b**	Describe any methods required to prepare the data for synthesis.	§2.4 (unit conversion to %BW; r = 0.5 for pre–post correlation)
**13c**	Describe any methods used to tabulate or visually display results.	§2.6 (forest plots; RevMan/CMA); §3 figures/tables
**13d**	Describe any methods used to synthesise results (model, heterogeneity, software).	§2.6 (random-effects DerSimonian–Laird; I^2/Cochran's Q; subgroup analysis; RevMan 5.4.1, CMA 3.0)
**13e**	Describe any methods used to explore heterogeneity (e.g., subgroup analysis).	§2.6 (subgroup by population type)
**13f**	Describe any sensitivity analyses.	§2.6; §3.6 (leave-one-out; low-RoB-only)
**14**	Describe any methods used to assess risk of bias due to missing results (publication bias).	§2.6 (funnel/Egger planned only if ≥10 studies; not performed)
**15**	Describe any methods used to assess certainty in the body of evidence.	§2.7 (GRADE)

RESULTS

**#**	**Item**	**Reported (Location)**
**16a**	Describe the results of the search and selection process (numbers).	§3.1; Figure 2 (PRISMA flow): 8,653 → 7,002 screened → 478 full-text → 8 publications (9 comparisons) included
**16b**	Cite studies that might appear to meet inclusion criteria but were excluded, with reasons.	§3.1 (full-text exclusion reasons, n = 469)
**17**	Cite each included study and present its characteristics.	§3.2; Table 1
**18**	Present assessments of risk of bias for each included study.	§3.3; Figure 1
**19**	Present for all outcomes, for each study: summary statistics and effect estimate with CI.	Table 1 (per-study Hedges' g [95% CI]); Figures 3–4
**20a**	For each synthesis, briefly summarise the contributing studies.	§3.4
**20b**	Present results of all statistical syntheses (estimate, CI, heterogeneity).	§3.4 (overall g = −0.94; subgroups); §3.5 (knee flexion)
**20c**	Present results of all investigations of possible causes of heterogeneity.	§3.4.2 (subgroup analysis)
**20d**	Present results of all sensitivity analyses.	§3.6
**21**	Present assessments of risk of bias due to missing results.	§3.7 (not assessed; <10 studies)

22	Present assessments of certainty for each outcome.	§3.8; Table 2 (GRADE — low)

DISCUSSION

#	Item	Reported (Location)
23a	Provide a general interpretation of the results in the context of other evidence.	§4 Discussion
23b	Discuss any limitations of the evidence included in the review.	§4.1 Limitations
23c	Discuss any limitations of the review processes used.	§4.1 Limitations
23d	Discuss implications for practice, policy, and future research.	§4, §5

OTHER INFORMATION

#	Item	Reported (Location)
24a	Provide registration information, including register name and number.	§2.1 (PROSPERO CRD42025116119)
24b	Indicate where the review protocol can be accessed.	§2.1 (PROSPERO link)
24c	Describe and explain any amendments to the protocol.	§2.1 (deviations: search date; outcome construct clarification; author list)
25	Describe sources of financial/non-financial support and role of funders.	Funding statement
26	Declare any competing interests.	Conflicts of Interest statement
27	Report which of the following are publicly available and where: data, code, other materials.	Data Availability Statement; Supplementary Materials

PRISMA 2020 = Preferred Reporting Items for Systematic Reviews and Meta-Analyses 2020. Reference: Page MJ, et al. BMJ 2021;372:n71.
