# Supplementary material for: Movement Retraining and Peak Landing Force, a Modifiable Anterior Cruciate Ligament Injury Risk Marker, in Athletes: A Systematic Review and Meta-Analysis for Primary Prevention
Source: J Funct Morphol Kinesiol. 2026 Jun 29;11(3):259. doi: 10.3390/jfmk11030259 (PMC13398292; doi:10.3390/jfmk11030259)
Supplement: Supplementary file 1 [file jfmk-11-00259-s001.zip › Table_S3_RoB2_assessment.pdf]

Table S3. Risk-of-bias (RoB 2) assessment per study

Movement Retraining and Peak Landing Force, a Modifiable Anterior Cruciate Ligament Injury Risk Marker, in Athletes: A Systematic Review and Meta-Analysis for Primary Prevention

Study	D1	D2	D3	D4	D5	Overall
**Lephart 2005**	Some concerns	Some concerns	Some concerns	Some concerns	Some concerns	Some concerns
**Vescovi 2008**	Some concerns	Low	Some concerns	Low	Some concerns	Some concerns
**Tate 2013**	Some concerns	Low	Low	Some concerns	Some concerns	Some concerns
**Hopper 2017**	Low	Low	Low	Low	Low	Low
**Zhao 2024**	Low	Low	Low	Low	Some concerns	Some concerns
**Iida 2013**	Some concerns	Some concerns	Low	Low	Low	Some concerns
**Ericksen 2016**	Low	Low	Low	Low	Low	Low
**Ericksen 2018**	Low	Some concerns	Low	Some concerns	Low	Some concerns

D1, randomisation process; D2, deviations from intended interventions; D3, missing outcome data; D4, measurement of the outcome; D5, selection of the reported result. Two reviewers (H.S. and Y.H.) assessed each study independently; disagreements were resolved by consensus.
