# Supplementary material for: Movement Retraining and Peak Landing Force, a Modifiable Anterior Cruciate Ligament Injury Risk Marker, in Athletes: A Systematic Review and Meta-Analysis for Primary Prevention
Source: J Funct Morphol Kinesiol. 2026 Jun 29;11(3):259. doi: 10.3390/jfmk11030259 (PMC13398292; doi:10.3390/jfmk11030259)
Supplement: Supplementary file 1 [file jfmk-11-00259-s001.zip › Table_S4_GRADE_evidence_profile.pdf]

Table S4. GRADE evidence profile

Movement Retraining and Peak Landing Force, a Modifiable Anterior Cruciate Ligament Injury Risk Marker, in Athletes: A Systematic Review and Meta-Analysis for Primary Prevention

Outcome	Comparisons (participants)	Risk of bias	Inconsistency	Indirectness	Imprecision	Publication bias	Hedges' g (95% CI)	Certainty
**Peak vGRF, all populations**	9 (292)	Serious (some concerns in 6/8 studies)	Serious (I^2 = 63%)	Not serious	Not serious	Not assessed (<10 studies)	−0.94 (−1.34, −0.54)	Low ⊕⊕○○
**Peak vGRF, pivot sport subgroup**	6 (194)	Serious (some concerns in 4/6 comparisons)	Not serious (I^2 = 45%)	Not serious	Serious (below optimal information size)	Not assessed (<10 studies)	−0.66 (−1.05, −0.26)	Low ⊕⊕○○

Both outcomes began at high certainty (randomised trials) and were downgraded two levels to low. Certainty was not upgraded for effect size. vGRF, vertical ground reaction force; CI, confidence interval. Two reviewers (YH.K. and H.S.) rated certainty independently.
